# Where the wild things are: genetic associations of environmental adaptation in the *Oryza rufipogon* species complex

**DOI:** 10.1093/g3journal/jkad128

**Published:** 2023-06-09

**Authors:** Diane R Wang, Michael B Kantar, Varunseelan Murugaiyan, Jeffrey Neyhart

**Affiliations:** Department of Agronomy, Purdue University, West Lafayette, IN 47907, USA; Department of Tropical Plant and Soil Sciences, University of Hawai’i at Manoa, Honolulu, HI 96822, USA; Rice Breeding Platform, International Rice Research Institute (IRRI), DAPO Box 7777, Metro Manila 1301, Philippines; USDA-ARS, Genetic Improvement for Fruits and Vegetables Laboratory, Chatsworth, NJ 08019, USA

**Keywords:** rice, crop wild relatives, diversity, environmental association, soil, temperature, precipitation

## Abstract

Crop wild relatives host unique adaptation strategies that enable them to thrive across a wide range of habitats. As pressures from a changing climate mount, a more complete understanding of the genetic variation that underlies this adaptation could enable broader utilization of wild materials for crop improvement. Here, we carry out environmental association analyses (EAA) in the *Oryza rufipogon* species complex (ORSC), the wild progenitor of cultivated Asian rice, to identify genomic regions associated with environmental adaptation characterized by variation in bioclimatic and soil variables. We further examine regions for colocalizations with phenotypic associations within the same collection. EAA results indicate that significant regions tend to associate with single environmental variables, although 2 significant loci on chromosomes 3 and 5 are detected as common across multiple variable types (i.e. precipitation, temperature, and/or soil). Distributions of allele frequencies at significant loci across subpopulations of cultivated *Oryza sativa* indicate that, in some cases, adaptive variation may already be present among cultivars, although evaluation in cultivated populations is needed to empirically test this. This work has implications for the potential utility of wild genetic resources in pre-breeding efforts for rice improvement.

“Let the wild rumpus start!”

*— Maurice Sendak, Where the Wild Things Are*

## Introduction

Crop wild relatives, from which the world's most essential plant species derive, have been subject to the forces of natural selection over millions of years. This has enabled their adaptation across diverse environments, and as a result, wild gene pools represent rich repositories of novel alleles that can be utilized for plant improvement (Dempewolf *et al*. 2014; Hübner and Kantar 2021). However, despite individual success stories of pre-breeding using inter-specific crosses (e.g. Zamir 2001; McCouch *et al*. 2007; Mammadov *et al*. 2018), investment in leveraging these exotic materials for complex traits has historically been limited. This can be attributed to the occurrence of linkage drag that arises from inter-specific crosses, which disrupts favorable gene complexes present in breeding gene pools. Accordingly, benefits of wild introgressions may not be fully revealed until after many rounds of backcrossing, substantially increasing the time and labor involved in utilizing wild material (Bohra *et al*. 2022).

In recent years, there has been increased efforts in using ex situ germplasm collections as sources of traits to help adapt crops to climate change (Byrne *et al*. 2018; Mascher *et al*. 2019). Different approaches have been suggested as the most expedient to identifying promising accessions, for example, Focused Identification of Germplasm Strategy (FIGS), which leverages environmental parameters describing collection sites as selection criteria (Khazaei *et al*. 2013). Other strategies include first identifying alleles underlying environmental adaptation (i.e. environmental association analysis, as reviewed by Rellstab *et al*. (2015). When georeferences of collection sites and historical climatic data are available, panels of crop wild relatives can enable identification of beneficial alleles for abiotic stress adaptation. Once these alleles have been identified, cultivated collections can be screened for individuals that carry these alleles, which may be used for crossing to limit the occurrence of linkage drag. This environmental association approach to identify loci and alleles of importance has been applied to collections of wild relatives of a wide range of crop species, for example, those of barley (Russell *et al*. 2016; Lei *et al*. 2019), common bean (Cortés and Blair 2018), soybean (Anderson *et al*. 2016), and cranberry (Neyhart *et al*. 2022). These studies were able to identify candidate genes for diverse abiotic stress such as drought and low temperature and dissect the genetic basis of local adaptation. Yet, no studies to our knowledge have examined the wild relatives of rice using environmental association.

The *Oryza rufipogon* species complex (ORSC) is the wild progenitor of cultivated Asian rice, *Oryza sativa*, a staple crop particularly important for feeding the world's poorest populations. The ORSC's geographic range is distributed widely across South, Southeast, and Eastern Asia, and the species occupies diverse tropical and subtropical habitats often found near or within human-disturbed areas such as cultivated fields, ditches, and irrigation channels (Vaughan *et al*. 2008). While historical classification defined separate perennial and annual species based on conventional morphological traits related to life history and mating habit (Sharma and Shastry 1965), recent genetic studies have not found conclusive evidence of this differentiation. Rather, genetic data support a single species comprised of diverse ecotypes (perennial, annual, and intermediate) and a high degree of admixture with cultivated *O. sativa* (Civan *et al*. 2015; Kim *et al*. 2016; Wang, Vieira, *et al*. 2017; Choi and Purugganan 2018; Eizenga *et al*. 2022).

Leveraging a previously genotyped and recently phenotyped collection of ORSC accessions (Kim *et al*. 2016; Eizenga *et al*. 2022), we take a genome-wide association approach to identify loci that underlie environmental adaptation across ORSC's native range in Asia. Specifically, our study aims to address the following questions: (1) Do loci associated with environmental adaptation generally correspond to multiple abiotic variables or to singletons? (2) Do loci associated with environmental adaptation colocalize with regions of the genome associated with phenotypes? And (3) are alleles of interest present among cultivated relatives? Our results have implications for the potential use of wild rice genetic resources for crop improvement under changing climates.

## Materials and methods

### Data sources

All data analyzed in this study were retrieved from public sources. Genotype data [113,739 single nucleotide polymorphisms (SNPs)] on a panel of 286 ORSC accessions were originally published by Kim *et al*. (2016), and a full description of the germplasm panel and genotyping methods may be found there. Briefly, the panel originated from 15 countries, sampling across the species distribution of South, Southeast, and Eastern Asia. Seeds were originally sourced from the International Rice Germplasm Collection (*n* = 283) in the Philippines and the National Institute of Genetics (*n* = 3) in Japan. Environmental data were sourced from WorldClim for precipitation and temperature variables (Fick and Hijmans 2017) and International Soil Reference and Information Centre (ISRIC) for soil variables (Hengl *et al*. 2015) for all ORSC accessions that were georeferenced; this resulted in a full dataset (i.e. having both genotype and environmental data) on 259 individuals. To account for the uncertainty in the geographic coordinates, we obtained the 19 bioclimatic variables at a resolution of 5 arcmin (approximately 47 km^2^) and the 10 soil variables at a resolution of 2.5 arcmin (approximately 12 km^2^). The soil data were available at 6 depths: 0–5, 5–15, 15–30, 30–60, 60–100, and 100–200 cm. These data were aggregated to 2 layers (topsoil and subsoil) using a weighted mean approach, whereby the weights were proportional to the size of the original layer depths (Anderson *et al*. 2016). A subset of the ORSC panel (*n* = 240) was recently phenotyped by Eizenga *et al*. (2022), and we also leveraged these data in the current study. Phenotypes consisted of Best Linear Unbiased Estimates (BLUEs) of the genotype mean of each line for 44 unique traits; for the current study, we utilized the BLUEs estimated from the site at IRRI.

### Network analysis

A network analysis was used to explore the relationships between environmental variables and phenotypic variables. First, a correlation matrix was constructed using the correlate function from the corrr library (Kuhn *et al*. 2020). This matrix was next transformed into a correlation network with a minimum correlation coefficient of *r* = 0.3. Communities of highly connected clusters of environmental and phenotypic variables were detected using group_infomap() in the R package ggraph (Pedersen *et al*. 2017). Briefly, soil, climate, and phenotype communities were identified using the spinglass algorithm, a function that detects communities in a graph defining a community as a set of nodes with many edges inside the community and few edges outside (i.e. between the community itself and the rest of the graph).

### Principal component analysis

Principal component analysis (PCA) on the 16 phenotypic variables that clustered with bioclimatic and biophysical variables, first standardized to a mean of 0 and standard deviation of 1, was conducted using the R library FactoMineR (Lê *et al*. 2008). These 16 variables were CULT—culm length, LIGLT—ligule length, DIST—distance of nearest spikelet to panicle base, DTHD—days to 50% heading, ANTLT—anther length, STGLT—stigma length, PNLG—panicle length, CUDI—culm diameter, X2LWD—penultimate leaf width, FLFWD—flag leaf width, UNFILLED—number of empty spikelets per panicle, X2LLT—penultimate (2nd) leaf length, FLFLG—flat leaf length, SPKWD—spikelet width, STLLT—sterile lemma length, and SPKLT—spikelet length. Scores for PCs 1–5 were then used as inputs for phenotype genome-wide association analyses (below).

### Genome-wide association analyses

Due to the sparse nature of Genotyping-By-Sequencing data, the SNP dataset was first filtered for missingness (≤0.15) and minor allele frequency (≥0.05) using Plink1.9 (Purcell *et al*. 2007). The resulting set of 14,302 SNPs (see Supplementary Fig. 1 for SNP distribution across genome) was retained to construct an additive relationship matrix (**K**) using the R package *rrBLUP* v. 4.6.1 (Endelman 2011).

Genomewide association (GWA) analyses were run using the GWAS() function in *rrBLUP* with environmental variables as phenotypes (19 bioclimatic variables and 10 soil variables × 2 layers = 39 total variables). GWA was also carried out using scores from the first 5 components (*n* = 5 variables) that resulted from the phenotype PCA. Using the *P*-value inflation factor (lambda), we initially compared 3 GWA models: naïve (no polygenic background effect or adjustment for population structure), K (polygenic background effect only), and K + Q (both polygenic background and adjustment for population structure using different numbers of SNP-based principal components). The K + Q model with 2 PCs was identified as the best model based on having a high median lambda value but low variance; this model was used for all subsequent analyses (Supplementary Fig. 2). For environmental GWA, significant SNPs were identified as those passing a false discovery rate (FDR) of 0.05 using the Benjamini–Hochberg method. For GWA using phenotype PC scores, significant SNPs were identified as those passing an FDR of 0.10 using the Benjamini–Hochberg method. For all GWA runs, quantile–quantile plots were assessed visually to determine whether to interpret results; Manhattan plots and QQ plots of GWA runs that passed diagnostics are found in Supplementary Figs. 3–15.

### Candidate gene analysis

Significant SNPs were used to assemble lists of predicted nearby genes based on physical location using the IRGSP1.0 genome annotation (https://ftp.ensemblgenomes.ebi.ac.uk/pub/plants/release-55/gff3/oryza_sativa/; last accessed 2022 December 12). We limited our search to those predicted genes within 150 kb of a given associated SNP. This threshold was based on the average distance at which linkage disequilibrium (LD) decayed to 0.2 across the 6 identified ORSC subpopulations (Kim *et al*. 2016). For each significant SNP, we extracted the list of nearby genes, their distances from these genes, and the gene descriptions (Supplementary Table 1). Plotting was conducted using ggplot2 (Wickham *et al*. 2011). Frequencies of minor alleles at QTL of interest were calculated in cultivated Asian rice, *O. sativa*, by querying the SNPseek database (https://snp-seek.irri.org/), which houses data on 3,000 cultivated rice genomes (Alexandrov *et al*. 2015; Wang *et al*. 2018).

## Results and discussion

### Variation of temperature, precipitation, and soils across the range of ORSC

Accessions of the ORSC panel examined in this study originated from 15 countries across tropical and subtropical climatic zones of South, Southeast, and Eastern Asia (Myanmar, Indonesia, Thailand, Laos, Vietnam, India, Bangladesh, Sri Lanka, Cambodia, Malaysia, Philippines, Taiwan, China, Nepal, and Papua New Guinea). Out of the 41 environmental variables (19 bioclimatic and 10 soil × 2 depths) explored from these georeferenced locations of the ORSC panel, we found that soil organic carbon and precipitation of the driest month/quarter (bio14/bio17) had the greatest dispersion based on the coefficient of variation, while soil bulk density, mean temperature of the warmest quarter (bio10), and mean temperature of the wettest quarter (bio8) were found to be the least variable (Supplementary Table 2). In general, precipitation variables had greater variation than temperature variables (average CV of 0.89 for precipitation metrics compared to 0.22 for temperature metrics). These geographical and environmental ranges suggest wide adaptation potential of the ORSC, especially with respect to different hydrological conditions.

### Genomewide environmental associations

**Fig. 1.**
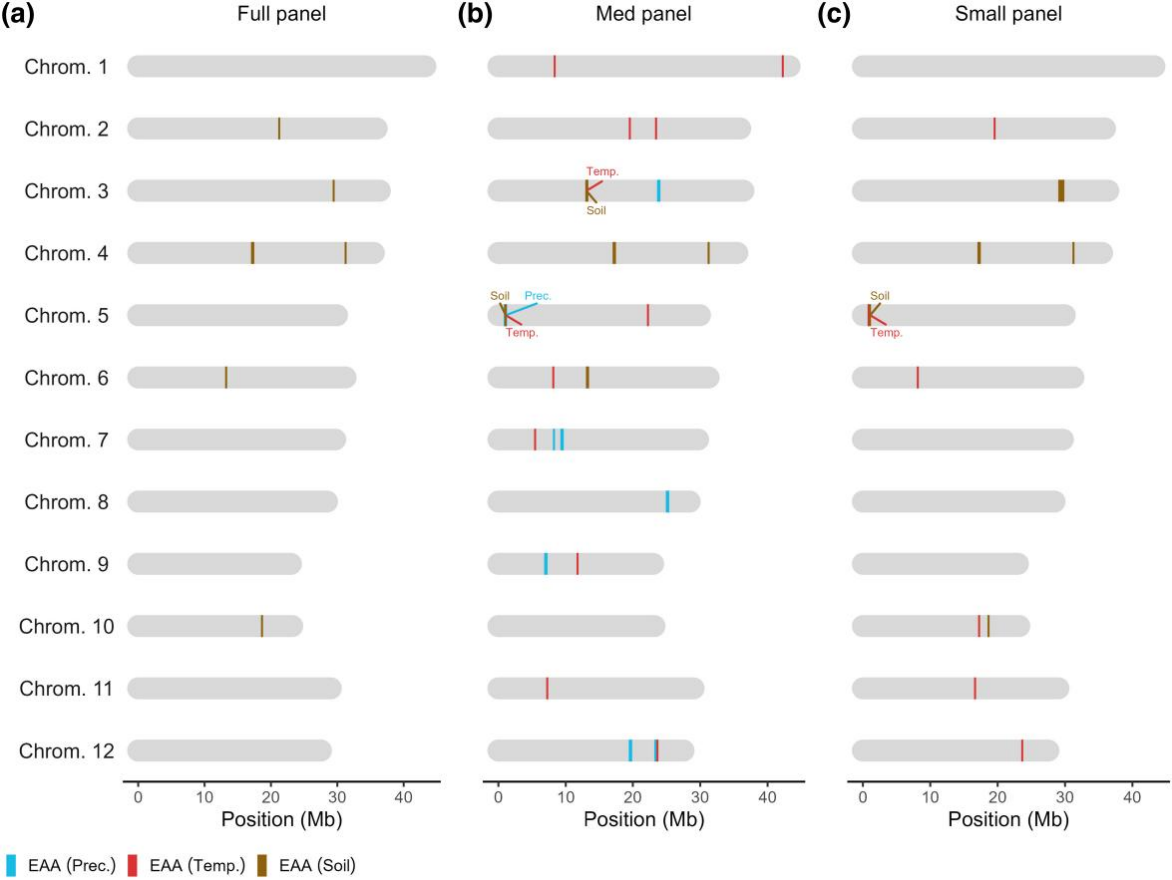
Summary of significant SNPs identified from genome-wide association analyses for environmental variables. Physical locations of significant SNPs are shown for the full (a), medium (b), and small (c) panel partitions. The medium panel partition yielded in the greatest number of significant SNPs across all 3 environmental variable types (precipitation, temperature, and soil), whereas the small and large partitions yielded in SNPs for only temperature and soil (small) or soil only (large). Co-localization of significant loci across environmental variable type are shown on chromosomes 3 and 5.

To identify regions of the genome associated with environmental adaptation across the geographical range of ORSC (South, Southeastern, and Eastern Asia), we carried out genome-wide association analyses on 19 bioclimatic variables and 10 soil variables, each at 2 depths, leveraging published genotype data and available georeference information on the panel. Previously, subpopulation-specific GWAS has been carried out with success in the highly stratified subpopulations of cultivated Asian rice, *O. sativa* (McCouch *et al*. 2016; Wang, Han, *et al*. 2017). While the population structure of the ORSC is much shallower than that of its cultivated relative, the W3 and W5 subpopulations are geographically constrained to Papua New Guinea and Nepal, respectively, and have been reported to be the most differentiated of the 6 subpopulations (W1–W6) identified out of the species complex (Kim *et al*. 2016). Therefore, we were interested to explore the impact of retaining or removing subpopulations in the panel on final GWA results. To this end, we analyzed 3 different panel partitions: the full panel (*n* = 259), a medium panel (*n* = 239, whereby individuals from the W3 subpopulation were removed), and a small panel (*n* = 229, whereby individuals of W5 and W3 were removed). Overall, we found that the medium panel yielded the most GWA runs that passed QC, resulting in 37 significant SNPs detected across 11 variables (2 precipitation, 2 temperature, and 7 soil), followed by the small panel, which yielded 16 significant SNPs detected across 2 temperature and 5 soil variables. The large panel, despite having the greatest number of individuals, only yielded 9 significant SNPs detected across 4 soil variables (Fig. 1 and Supplementary Table 3). This may be due to yet unaccounted-for population structure in the full panel when analyzing certain environmental variables that are highly differentiated in the isolated island region of Papua New Guinea; indeed, W3 stood out from the rest of the ORSC for several environmental variables, especially those related to precipitation [e.g. precipitation of the driest quarter (bio17), precipitation of the driest month (bio14), and precipitation seasonality (bio15)] and soil properties (e.g. soil organic carbon and bulk density) (Supplementary Fig. 16). Because the medium panel partition yielded the greatest number of significant loci and was the only partition that detected associations across all 3 environmental variable types (precipitation, temperature, and soil), we focused downstream analyses of GWA results from this panel.

Thirty-seven marker–environment associations were detected in the medium panel, which were distributed across all chromosomes except chromosome 10. These SNPs were associated with 2 precipitation variables, 2 temperature variables, and 7 soil variables, and comprised 22 unique genomic regions (i.e. significant SNPs at least 150 kb apart; see *Materials and Methods*). Chromosomes 7 and 12 harbored 3 significant loci each, chromosomes 1–6 and 9 contained 2 significant loci each, while chromosomes 8 and 11 had one each (Supplementary Table 3). We detected one potential QTL cluster, whereby adjacent significant SNPs identified for different environmental variables were not in LD of each other based on their own physical locations, however, could still be tagging the same causal gene due to some overlap in their LD windows. This was observed on chromosome 12, where a SNP at 23,635,879 bp was significant for a temperature variable [isothermality (bio3)], and another SNP ∼190 kb upstream located at 23,442,132 bp was detected for 2 closely related precipitation variables [precipitation of the driest month (bio14) and precipitation of the driest quarter (bio17)] (Fig. 1b). This region was located near a previously identified QTL on chromosome 12 at 23,250,434 bp that was associated with harvest index in cultivated diverse *indica* and *aus* rice (Bhandari *et al*. 2020).

Analyzing candidate genes for these adjacent QTLs on chromosome 12, there were a total of 16 gene models, of which 9 were named, that localized in the overlapped region of interest. Among these, there was one R2R3-type MYB transcription factor (*OsMYB91*) previously shown to be inducible by abiotic stress in rice (Zhu *et al*. 2015). While salinity stress was the primary focus of that previous study, *OsMYB91* expression was also induced by heat shock, drought, and polyethylene glycol. In addition to the MYB in this region of interest, there was a tandem array of 3 genes (*OsMT1a*, *OsMT1g*, and *MT14C*) that encode for MT-like type 1 proteins. MTs in plants play known roles in the scavenging of reactive oxygen species (ROS). In rice, dehydration has been demonstrated to induce expression of an MT type-1 gene, and overexpression of the same gene improved drought tolerance (Yang *et al*. 2009). While the induction of MTs gene expression by temperature extremes, which would be directly related to isothermality, has not been demonstrated in rice, expression of metallothionein genes in other plant species are capable of being induced by a variety of abiotic stresses, including low temperature and drought (Kumar *et al*. 2012; Kim *et al*. 2014). Previous work has also demonstrated that the down-regulation of rice metallothionein type-1 genes leads to ROS accumulation and is necessary for aerenchyma formation upon waterlogging conditions (Yamauchi *et al*. 2017). These reported roles of *OsMYB91* and MT genes in rice and other plant species in mediating response to abiotic signals provide support that these genes could be responsible for the isothermality and precipitation QTLs identified on chromosome 12.

### Colocalizations of significant loci associated with precipitation, temperature, and soil variables

**Fig. 2.**
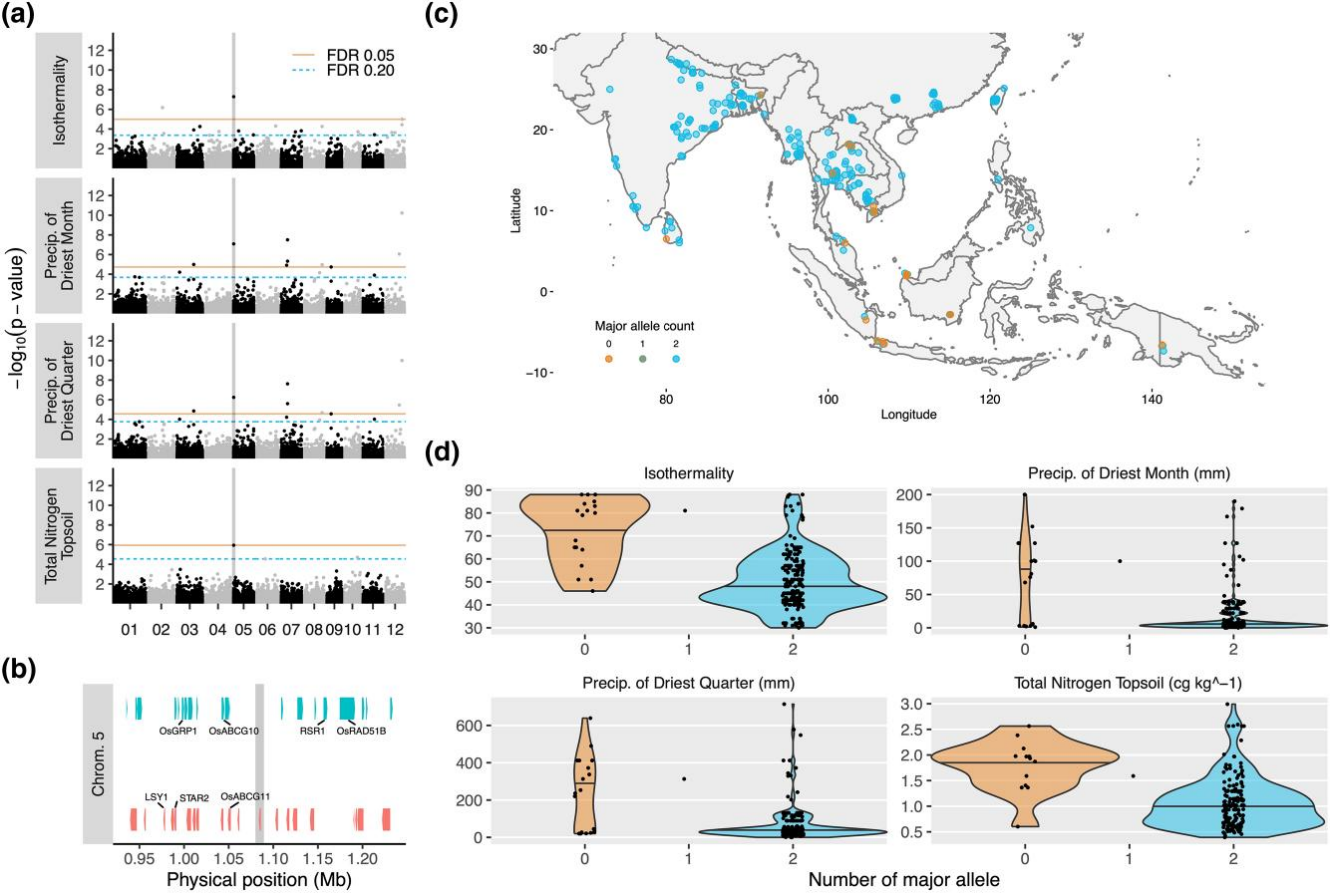
A locus on chromosome 3 is significant for temperature and soil variables. a) Manhattan plots of mean temperature of warmest quarter, pH of the subsoil, and pH of the topsoil. A SNP located on chromosome 3 at position 13,114,933 bp (vertical grey line) is found to be significant for all 3 environmental variables. b) Position of the significant SNP among gene models (MSUv7). There are a total of 34 gene models within 150 kb of the SNP, including 15 named genes (annotated). c) Geographical distribution of major and minor alleles across Asia and Oceania. d) Violin plots showing the distribution of values for the 3 environmental variables with respect to the major and minor allele at the significant SNP.

We next examined SNPs that were significant for 2 or more different types of environmental variables (i.e. precipitation, temperature, and soil). In addition to 10 SNP colocalizations identified between highly similar environmental variables [e.g. clay in topsoil with clay in subsoil or precipitation of the driest month (bio14) with precipitation of the driest quarter (bio17)] that would be expected, we detected 2 SNPs that colocalized across precipitation, temperature, and/or soil variables. One was located on chromosome 3 at 13,114,933 bp and was shared between one temperature variable [mean temperature of the warmest quarter (bio10)] and 2 related soil variables (pH of the topsoil and pH of the subsoil) (Fig. 2a). Within this region, there were 34 gene models, 15 of which were named genes (Supplementary Table 1; Fig. 2b). While none of the 15 named genes had obvious roles in integrating varied responses to multiple environmental signals, there were several transcription factors (*OsCW-ZF3* and *OsRFP*) as well as a superoxide dismutase (*SODCC1*), whose expression has been reported to be inducible by cold temperatures and ABA (Lee *et al*. 2009). Accessions harboring the minor allele of this significant SNP on chromosome 3 originated from regions that were warmer during the hottest quarter of the year (average 30.8°C vs average 28.4°C) and had less acidic soils (average 7.4 vs average 6.0 in the topsoil; average 6.5 vs average 5.3 in the subsoil) (Fig. 2, c and d). As soil pH is known to affect nutrient availability as well as metal toxicity, variation at this allele may play some role involving these conditions; however, the interaction with soil water also presents additional complexities for which we do not have information to speculate further.

**Fig. 3.**
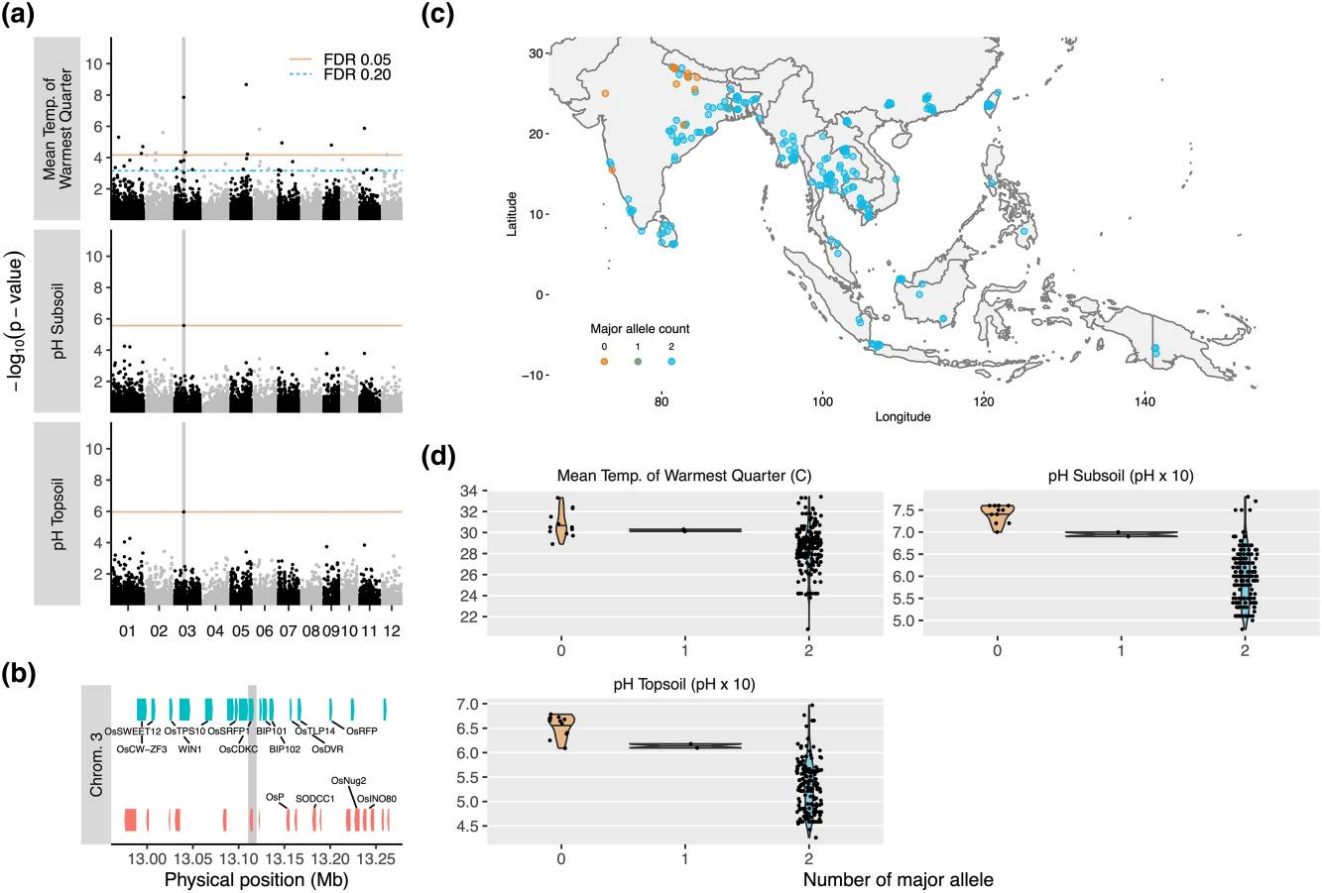
A locus on chromosome 5 is significant for temperature, precipitation, and soil variables. a) Manhattan plots of isothermality, precipitation in the driest month, precipitation of the driest quarter, and total nitrogen in topsoil. A SNP located on chromosome 5 at position 1,084,741 bp (vertical grey line) is found to be significant for all 4 environmental variables. b) Position of the significant SNP among gene models (MSUv7). There are a total of 41 gene models within 150 kb of the SNP, including 7 named genes (annotated). c) Geographical distribution of major and minor alleles across Asia and Oceania. d) Violin plots showing the distribution of values for the 4 environmental variables with respect to the major and minor allele at the significant SNP.

A second SNP of significance was detected on chromosome 5 at position 1,084,741 bp that colocalized between one temperature variable [isothermality (bio3)], one soil variable (nitrogen in the topsoil), and 2 precipitation variables [precipitation of the driest month (bio14) and precipitation of the driest quarter (bio17)] (Fig. 3a). These colocalized regions across different environmental variables were of particular interest for follow-up analysis, as they could point to genes with pleiotropic effects. We noted 41 gene models in this region that were annotated by IRGSP1.0 (Supplementary Table 1). Of 7 named genes, there was one homeobox gene that is important in leaf patterning (*LSY1*; Honda *et al*. 2018), a starch biosynthesis gene that affects seed development (*RSR1*; Fu and Xue 2010), and other genes that are involved in basic cellular processes (e.g. *OsRAD51B* involved in DNA repair). Also among the named genes were 3 ATP-binding cassette (ABC) proteins: one was a cloned gene involved in aluminum tolerance (STAR2; Huang *et al*. 2009) and the other 2 were ABC subgroup G transporters (*OsABCG10* and *OsABCG11*). Found in both prokaryotes and eukaryotes, ABC proteins are primarily transmembrane transporters that also contain a conserved nucleotide binding domain. In plants, these transporters are documented to play varied and critical roles in cellular detoxification (e.g. of heavy metals), growth and development (e.g. hormone transport and cuticle development), and pathogen defense (e.g. via secretion of plant secondary metabolites) (Kretzschmar *et al*. 2011). Diversification of these transporters are suggested to have enabled plants' highly plastic responses to environmental signals, a necessity for success as sessile organisms (Kretzschmar *et al*. 2011). In rice, *OsABCG10* and *OsABCG11* on chromosome 5 have been suggested to result from tandem duplication, with *OsABCG10* being a pseudogene based on lack of expression (Matsuda *et al*. 2012). In contrast, expression of *OsABCG11* has been shown to respond to temperature (up-regulation in both shoot and root after cold treatment; up-regulation in shoot after heat treatment) and various phytohormones such as abscisic acid (down-regulation in root), gibberellic acid (down-regulation in root), and jasmonic acid (up-regulation in both shoot and root) (Matsuda *et al*. 2012). Examining the environmental data, we observed that accessions harboring the minor allele of the significant SNP at this QTL originated from regions that tended to be wetter [average of 71 vs 15 mm for precipitation in the driest month (bio14); average of 238 vs 66 mm for precipitation in the driest quarter (bio17)], contained greater levels of nitrogen in the topsoil (average of 1.79 vs 1.07 cg kg^−1^), and had higher diurnal temperature fluctuations relative to annual temperatures [average of 72.4 vs 49.9% for isothermality (bio3)] (Fig. 3). Based on the diverse roles played by ABC transporters in plant response to abiotic signals, *OsABCG11* is a plausible candidate gene for the QTL identified on chromosome 5 associated with precipitation, temperature, and soil variation.

### Genome-wide associations of wild rice phenotypes

**Fig. 4.**
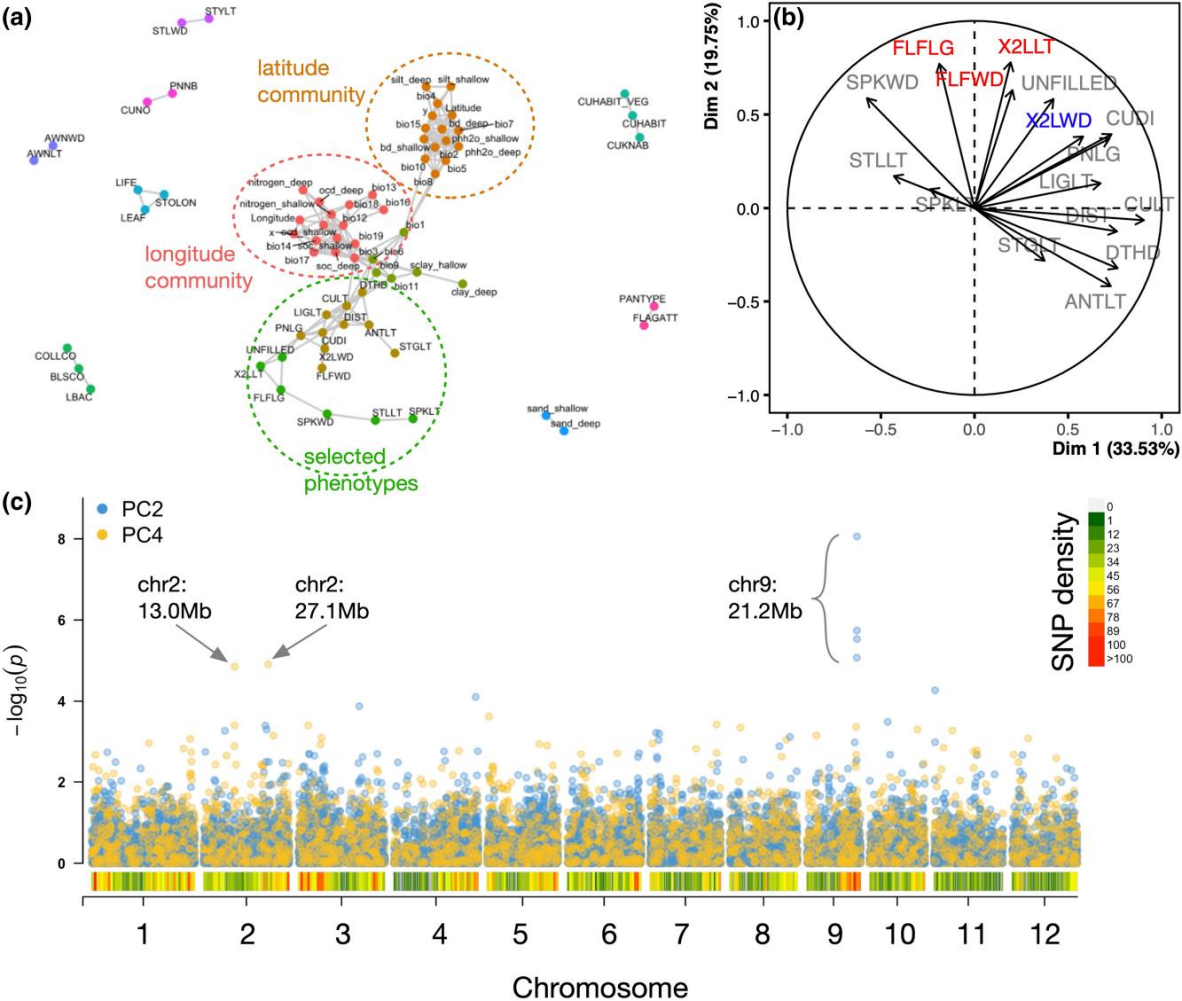
Genome-wide associations of select phenotypes in wild rice. a) Results of correlation network analysis and community detection using environmental and phenotypic data. Different communities are denoted as different colors. A total of 16 phenotypes clustered most with longitude (red) and latitude (orange): brown cluster (CULT, culm length; LIGLT, ligule length; DIST, distance of nearest spikelet to panicle base; DTHD, days to 50% heading; ANTLT, anther length; STGLT, stigma length; PNLG, panicle length; CUDI, culm diameter; X2LWD, penultimate (2nd) leaf width; FLFWD, flag leaf width) and the green cluster (UNFILLED, number of empty spikelets per panicle; X2LLT, penultimate (2nd) leaf length; FLFLG, flat leaf length; SPKWD, spikelet width; STLLT, sterile lemma length; SPKLT, spikelet length). These 16 phenotypes were selected for further analyses. b) The contribution of variables from Principal Components Analysis using the 16 selected phenotypes measured on 222 *O. rufipogon* accessions. Variables that had correlations of >0.60 with PC2 and PC4 (the PCs for which significant genomic associations were found) are FLFLG, FLFWD, X2LLT (PC2) and X2LWD (PC4) (Supplementary Table 4). c) Manhattan plot showing genome-wide association analysis results using the scores from PC2 and PC4. Annotated loci on chromosomes 2 and 9 are from markers that were found significant at a false discovery rate threshold of 0.05 by the Benjamini–Hochberg method.

To investigate whether loci significant for environmental variables also colocalized with regions of the genome associated with plant phenotypes, we leveraged trait data published recently on this panel by Eizenga *et al*. (2022). We first subsetted the dataset to include only those that were collected in a common garden experiment under screenhouse conditions in Los Baños, Philippines (14.1678°N, 121.2545°E), with the rationale that this location (in contrast to Arkansas, USA or New York, USA, the 2 other sites where data were collected) was located nearest to the native range of the ORSC. Phenotypes included several morphological traits at various scales (e.g. seed length/width; leaf length/width), phenology (e.g. days to heading), classical yield components (e.g. panicles per plant), and metrics related to wild rice ecotypes (e.g. rhizome and stolon formation; seed shattering). A network analysis was used to explore the relationships between these 44 phenotypic variables and the 41 environmental variables (Fig. 4a). We observed a primary cluster containing 5 communities, which included all the environmental variables and 16 of the phenotypes (Fig. 4b). Outside of this primary cluster were smaller (2- to 3-member) satellite communities containing only plant trait variables. Surprisingly, all of the ecotype-related traits, which we would have expected to cluster with environmental variables, fell out as part of the smaller satellite communities. We speculate that this is due to the spatial scale at which the environmental data were derived: while adaptive plant traits may be most reflective of an organism's more immediate environment (for example, a seasonally dry gully that could support annual but not perennial wild rice ecotypes), the bioclimatic and soil variables explored in our environmental GWAS captured variation at larger spatial scale. The other possibility is that the traits collected are not adaptive and therefore should not be expected to give rise to QTL that overlap with environmental associations.

To reduce dimensionality of the phenotype dataset, a principal components analysis was carried out on the 16 phenotypes that did cluster with environmental variables. The first 5 principal components explained 78% of the total variation, and scores of these PCs were used as composite traits in conjunction with the SNP data to carry out genome-wide scans for associations. Results from GWA on scores from 2 of the components, PC2 and PC4, passed QC and yielded significantly associated SNPs (Fig. 4c). We found that the major phenotypic contributors to PC2 and PC4 were leaf morphometrics of either the flag leaf (i.e. the last leaf to emerge prior to flowering) or the penultimate (2nd upper-most) leaf (Supplementary Table 4). PC2 was associated with one locus on chromosome 9 tagged by 4 significant SNPs (around 21,212,206 bp) and PC4 was associated with 2 loci on chromosome 2, each tagged by 2 SNPs, around 13,030,761 and 27,153,172 bp (Supplementary Table 4). None of these 3 loci colocalized with the regions we detected that were associated with environmental variables, possibly due to lack of mechanistic basis for flag and penultimate leaf morphology having a role with adaptation across environments. However, we note that association analysis of these composite traits yielded stronger and more significant loci than any association analysis on these same univariate phenotypes, including those beyond the 16 selected.

### Allele frequencies across wild and cultivated subpopulations

We next cataloged the frequency of the minor alleles at the QTLs identified on chromosomes 3 and 5 across the W1, W2, W4, W5, and W6 subpopulations of ORSC as well as in its cultivated relative, *O. sativa*. Except for the W5 subpopulation for the QTL on chromosome 3, the 2 minor alleles were rare in the wild populations (0–0.2). In contrast, we observed very high frequencies in the cultivated populations based on a database of 3,000 re-sequenced rice genomes (*Materials and Methods*). In the cultivated species, *O. sativa*, the frequency of the minor allele for the QTL on chromosome 5 was 0.67 in the *tropical japonica* subpopulation and fixed or nearly fixed in all other subpopulations while the minor allele of the chromosome 3 QTL was fixed across cultivated subpopulations (Table 1). Previous work analyzing these re-sequencing data on the 3,000 cultivated genomes had further classified the *indica* subpopulation into 3 sub-subpopulations (*indica-1*, *indica-2*, and *indica-3*), which were geographically associated (Wang *et al*. 2018). Examining allele frequencies at these smaller classes, we observed that the minor allele in chromosome 5, which was at 0.95 frequency overall in *indica* (Table 1), was lowest in *indica-3* (at 0.77); this population was localized to South Asia (vs China and Southeast Asia for *indica-1* and *indica-2*, respectively). We note, however, that the presence of these alleles in cultivated populations does not necessarily indicate they have the same effects as in wild populations. More experimental work in *O. sativa* is needed to test this.

**Table 1.** Frequency of minor allele across wild and cultivated subpopulations.

	Wild rice						Cultivated rice				
	W1	W2	W4	W5	W6	Admix	tej	trj	aro	ind	aus
chrom 5 SNP	0.2	0	0	0	0	0.2	0.99	0.67	1	0.95	1
chrom 3 SNP	0	0	0	0.82	0	0.06	1	1	1	1	1

Frequency in the W3 ORSC population is not shown due to its exclusion from the medium panel GWA.

tej, temperate japonica; trj, tropical japonica; aro, aromatic; ind, indica; aus, aus.

The contrast between wild and cultivated subpopulation allele frequencies gives rise to the question of whether the presence of these minor alleles in the wild are due to gene flow from cultivated populations. *Oryza sativa* is sympatric with the range of the ORSC, and wild accessions are often found within- or near-cultivated fields (Vaughan *et al*. 2008). Accordingly, genetic evidence points to substantial admixture between the ORSC and *O. sativa* (Civáň *et al*. 2015; Kim *et al*. 2016; Wang, Vieira *et al*. 2017). Previous work on the same wild panel examined in the current study reported that W1 and W6 are the most frequently found populations that are admixed with *O. sativa* and that accessions from these subpopulations often carried domestication-related alleles (Kim *et al*. 2016). This would be consistent for the case of the minor allele of the chromosome 3 QTL, which is nearly fixed in *O. sativa* but found at 20% in W1; however, this would not explain the very high frequency of the minor allele for S5_1084741 in W5 (82%). Nonetheless, back-introgression may still be the most plausible explanation. The W5 subpopulation is small, geographically constrained in the mountainous region of Nepal, and highly differentiated at a whole-genome level from the *O. sativa* (Kim *et al*. 2016). The alternative hypothesis that this allele originally derived from standing variation in this isolated wild subpopulation and propagated throughout all the cultivated subpopulations is therefore unlikely. Occurrence of the reciprocal process remains to be tested, which could be carried out using haplotype analyses or examination of Fst values to determine the degree of differentiation between W5 and the cultivated populations in this localized region.

## Conclusion

Identification of genomic loci and alleles associated with environmental adaptation in crop wild relatives like the ORSC can help inform strategies of how to leverage these underutilized genetic resources for crop improvement under changing climates. Here, we find genomic regions associated with multiple environmental variable types (soil, precipitation, and temperature) detected in wild rice and discuss potential candidate genes within LD of their significant SNPs. Documentation of allele frequencies at these SNPs across the subpopulations of cultivated *O. sativa* helps provide a more comprehensive picture of how these markers could be leveraged in crop breeding programs, as favorable variation may already be found in certain cultivated groups. We note, however, that loci identified here through the genome-wide association approach detects tag-SNPs, and that it is possible that the causal variant may not be found in cultivated relatives, depending on the evolutionary trajectory of the regions of interest. As GWA is a means to cast a wide net to shortlist loci of interest, further experimentation will be needed to validate their effects and to identify functional genes, for example, using systematically developed inter-specific populations [e.g. those developed recently by Singh *et al*. (2020)]. Analyses like the ones presented in the current study can help shed light on possible paths forward towards broader utilization of wild rice genetic materials while building upon the valuable public resources developed through previous community efforts.

## Supplementary Material

jkad128_Supplementary_Data

## Data Availability

The original sources for all data used here are described within *Materials and Methods*. The extracted data used directly for analyses along with Supplementary Figs. 1–16 and Supplementary Tables 1–4 may be found at https://doi.org/10.5281/zenodo.7999743.
