# Supplementary material for: Where the wild things are: genetic associations of environmental adaptation in the Oryza rufipogon species complex
Source: G3 (Bethesda). 2023 Jun 9;13(8):jkad128. doi: 10.1093/g3journal/jkad128 (PMC10411557; doi:10.1093/g3journal/jkad128)
Supplement: jkad128_Supplementary_Data [file jkad128_supplementary_data.zip › Supplemental Figures envGWAS_revision_230502.pdf]

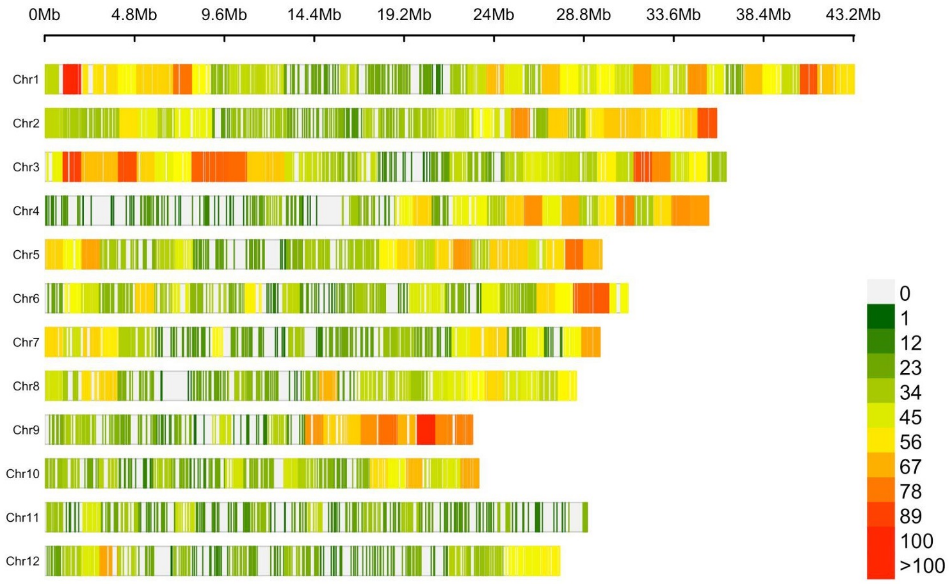

Figure S1. Distribution of SNPs across the genome. Color scale indicates SNP density across windows of 1 MB.

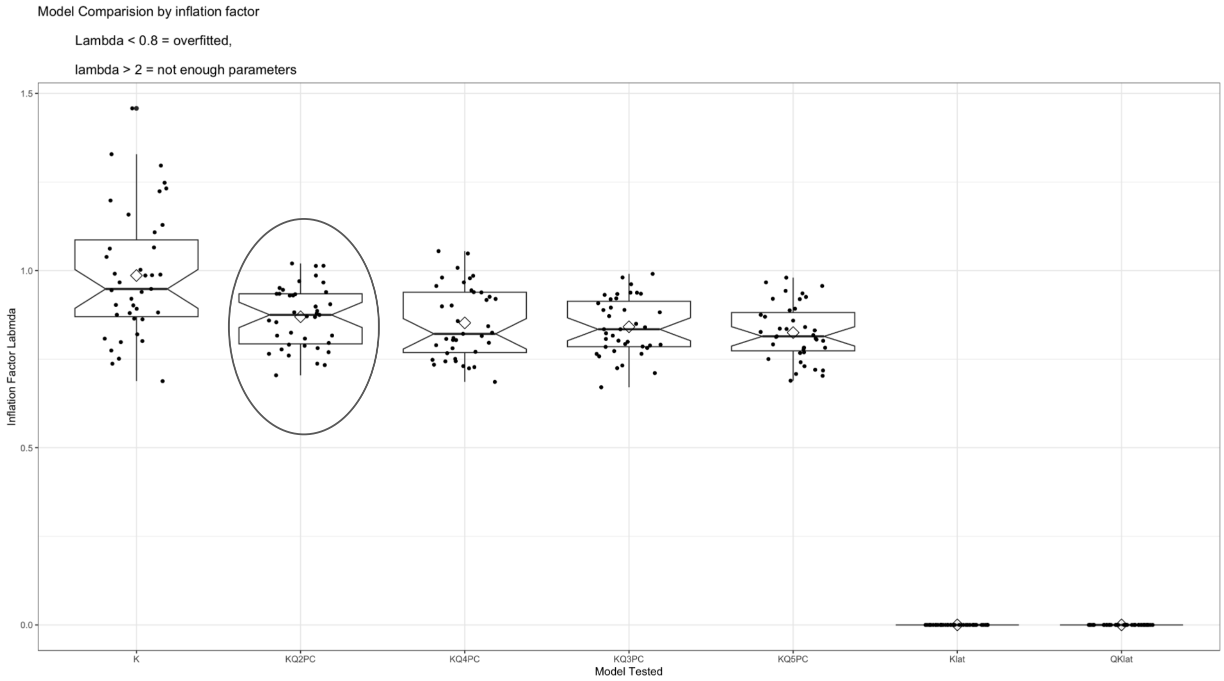

Figure S2. Model selection. Genomic inflation factor, lambda, was computed for each of the models tested. Going across the models are: K model, K + Q model with 2 PCs, K + Q model with 4 PCs, K + Q model with 3 PCs, K + Q model with 5 PCs, K model with latitude as a covariate, and finally, the K + Q model using 2 PCs with latitude as a covariate. The K + Q model with 2 PCs (circled) was selected for final GWA runs as it had the highest median lambda alon growth relatively low variance.

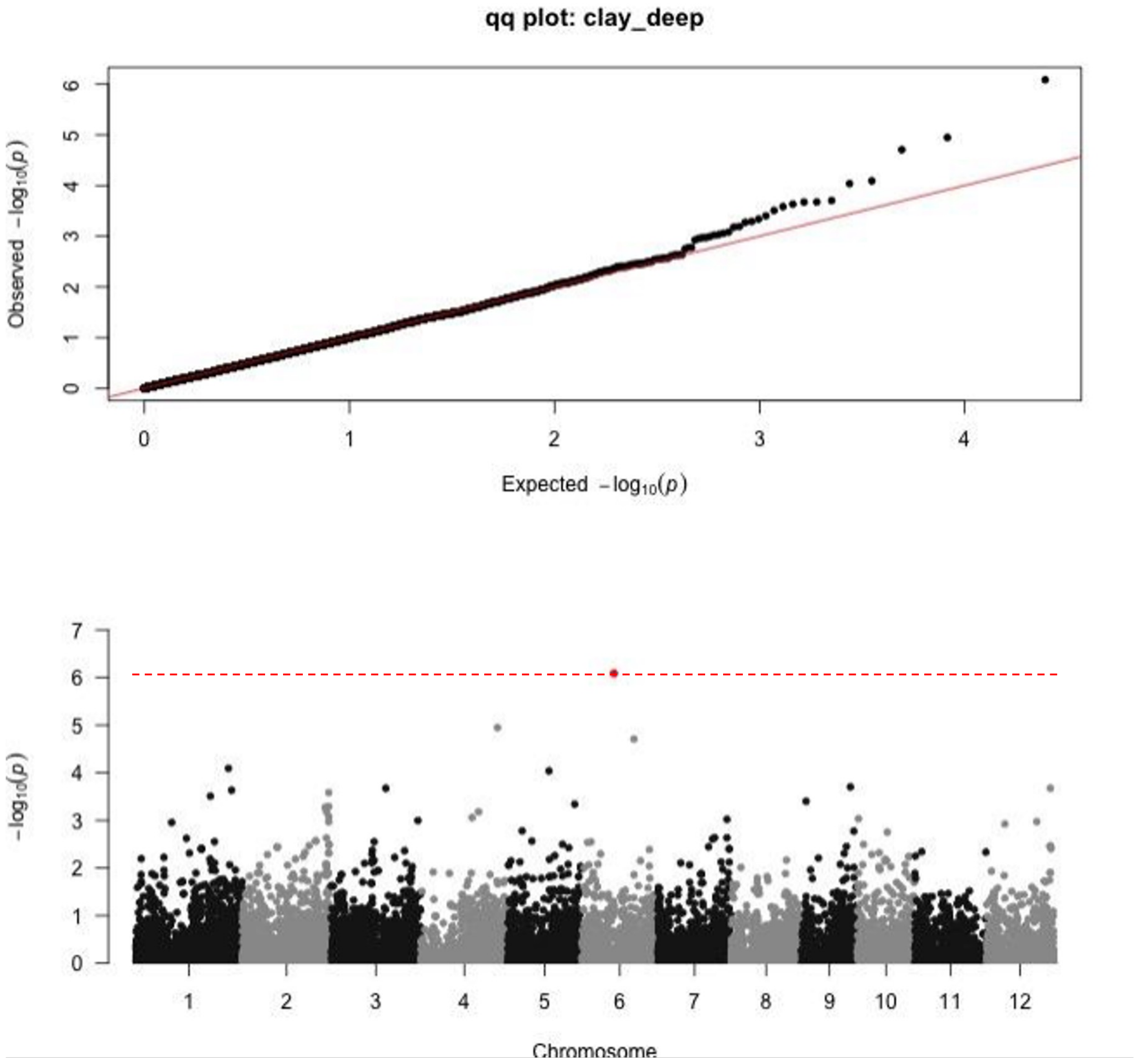

Figure S3. Genome-wide association results of clay (subsoil) in the medium panel. Shown are the quantile-quantile plot (top) and manhattan plot (bottom). Significant SNPs at False Discovery Rate threshold of 0.05 (Benjamini-Hochberg method) are annotated in red and a dashed red horizontal line is drawn at the level of the least significant SNP.

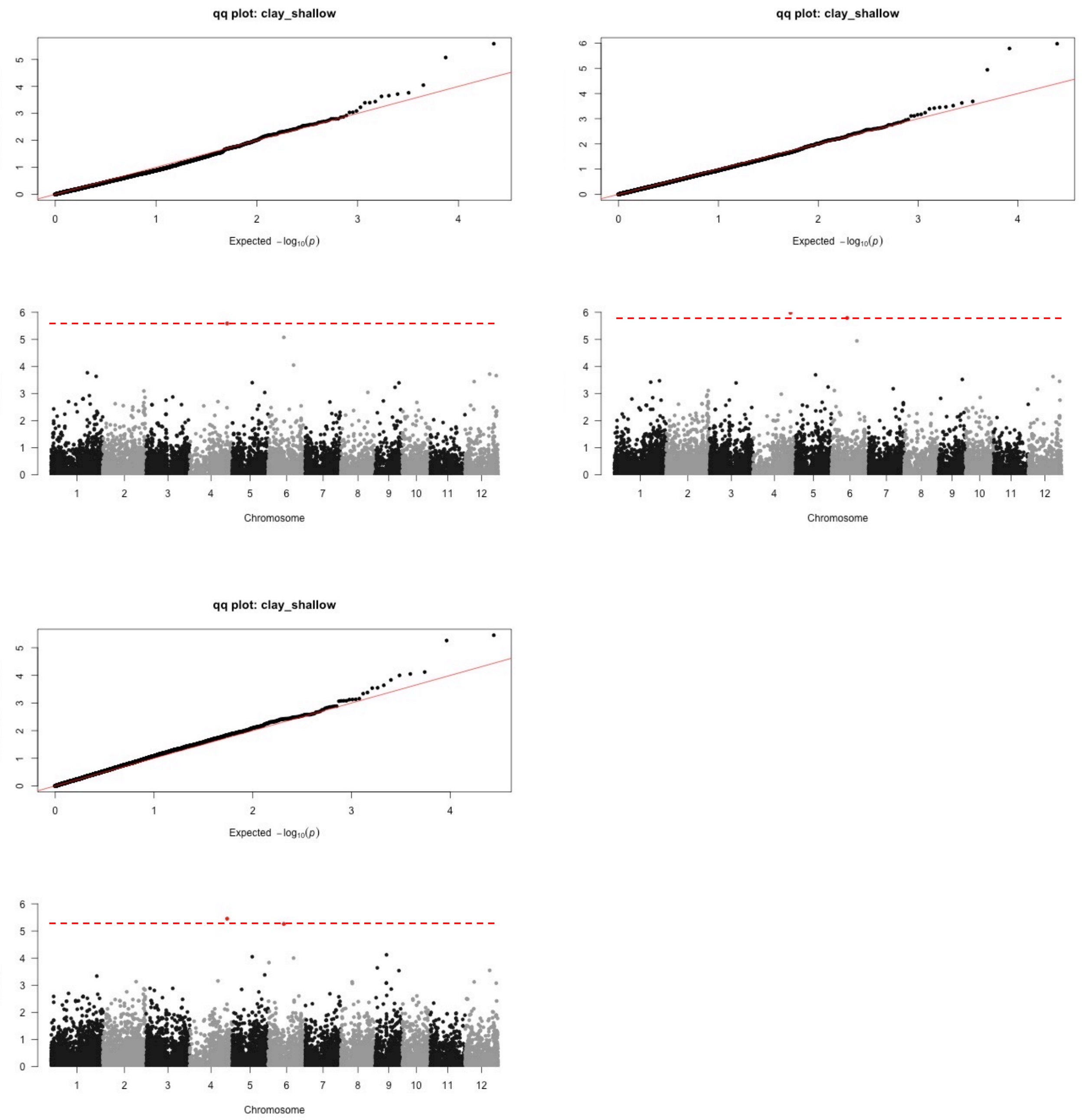

Figure S4. Genome-wide association results of clay (topsoil) in the small, medium, and full panels. From top-left to top-right to bottom-left: small, medium, and full. Shown are the quantile-quantile plot (top) and manhattan plot (bottom). Significant SNPs at False Discovery Rate threshold of 0.05 (Benjamini-Hochberg method) are annotated in red and a dashed red horizontal line is drawn at the level of the least significant SNP.

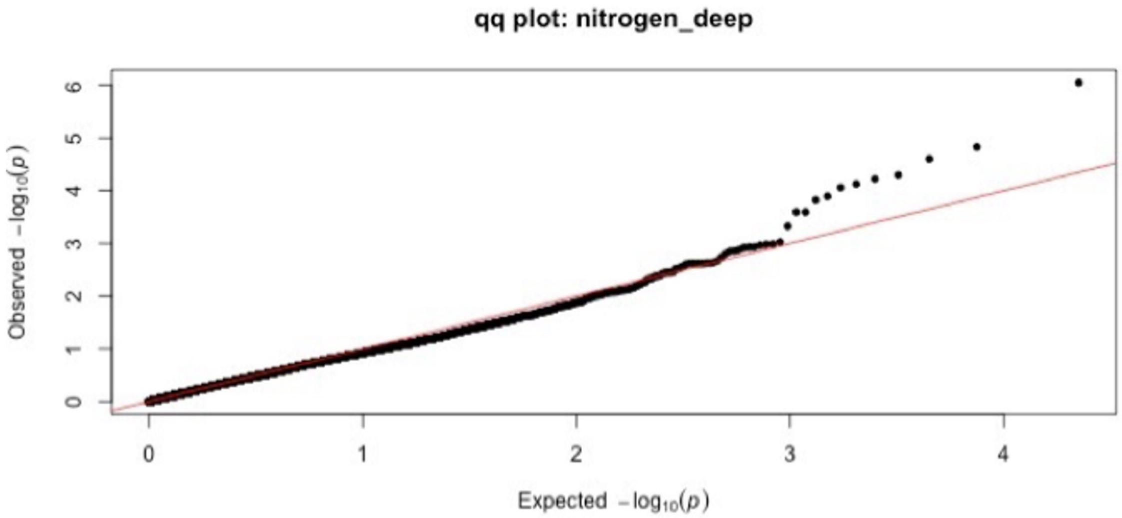

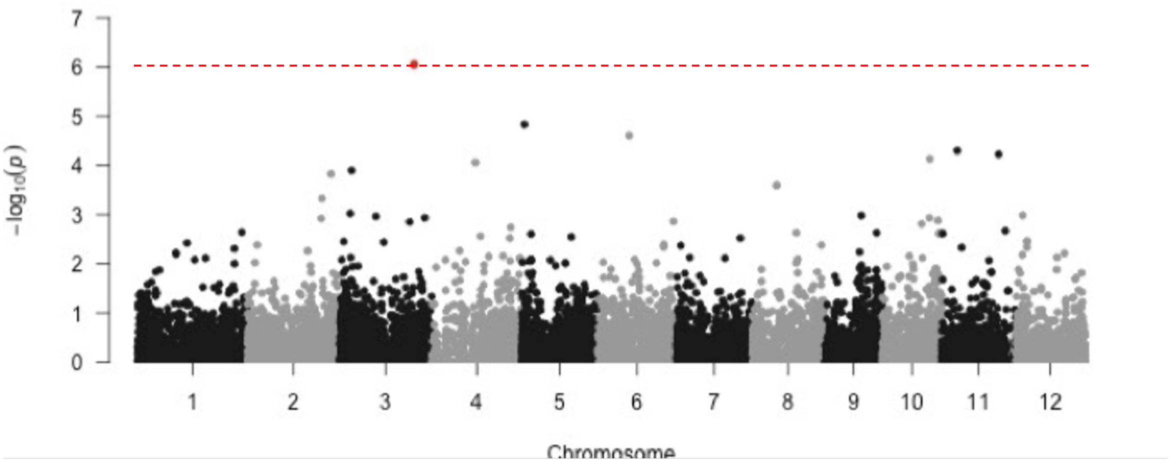

Figure S5. Genome-wide association results of nitrogen (subsoil) in the small panel. Shown are the quantile-quantile plot (top) and manhattan plot (bottom). Significant SNPs at False Discovery Rate threshold of 0.05 (Benjamini-Hochberg method) are annotated in red and a dashed red horizontal line is drawn at the level of the least significant SNP.

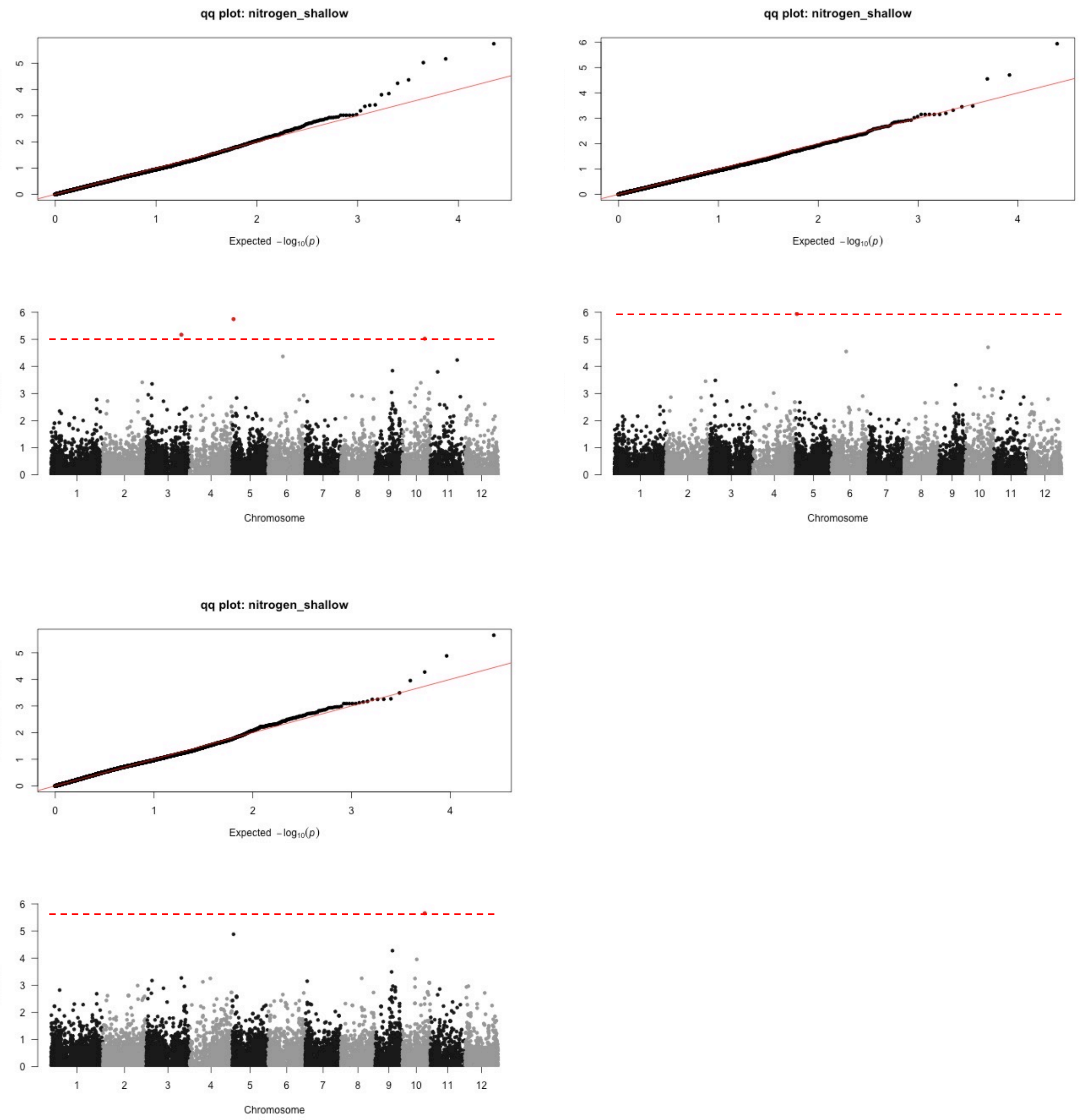

Figure S6. Genome-wide association results of nitrogen (topsoil) in the small, medium, and full panels. From top-left to top-right to bottom-left: small, medium, and full. Shown are the quantile-quantile plot (top) and manhattan plot (bottom). Significant SNPs at False Discovery Rate threshold of 0.05 (Benjamini-Hochberg method) are annotated in red and a dashed red horizontal line is drawn at the level of the least significant SNP.

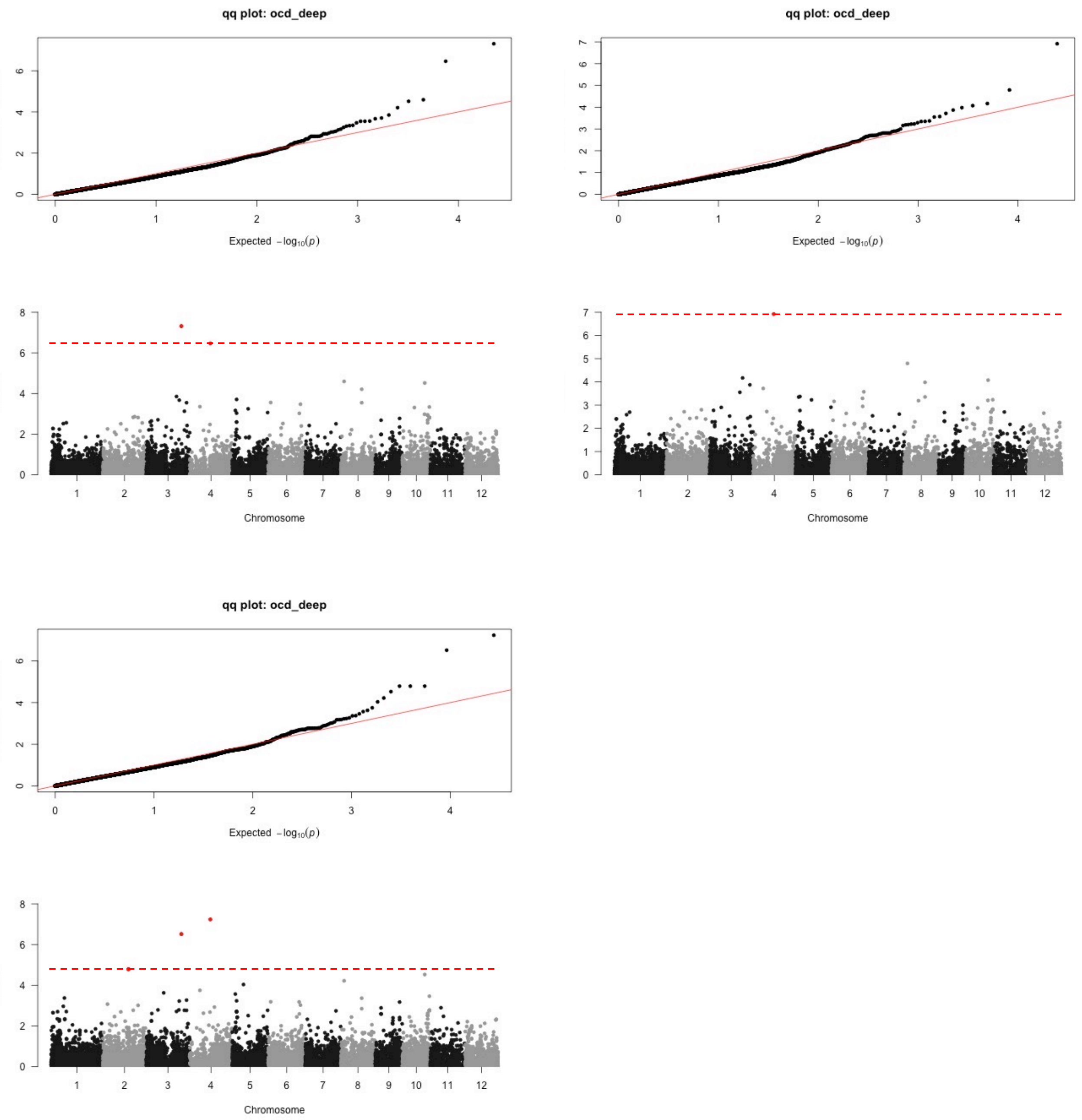

Figure S7. Genome-wide association results of organic carbon density (subsoil) in the small, medium, and full panels. From top-left to top-right to bottom-left: small, medium, and full. Shown are the quantile-quantile plot (top) and manhattan plot (bottom). Significant SNPs at False Discovery Rate threshold of 0.05 (Benjamini-Hochberg method) are annotated in red and a dashed red horizontal line is drawn at the level of the least significant SNP.

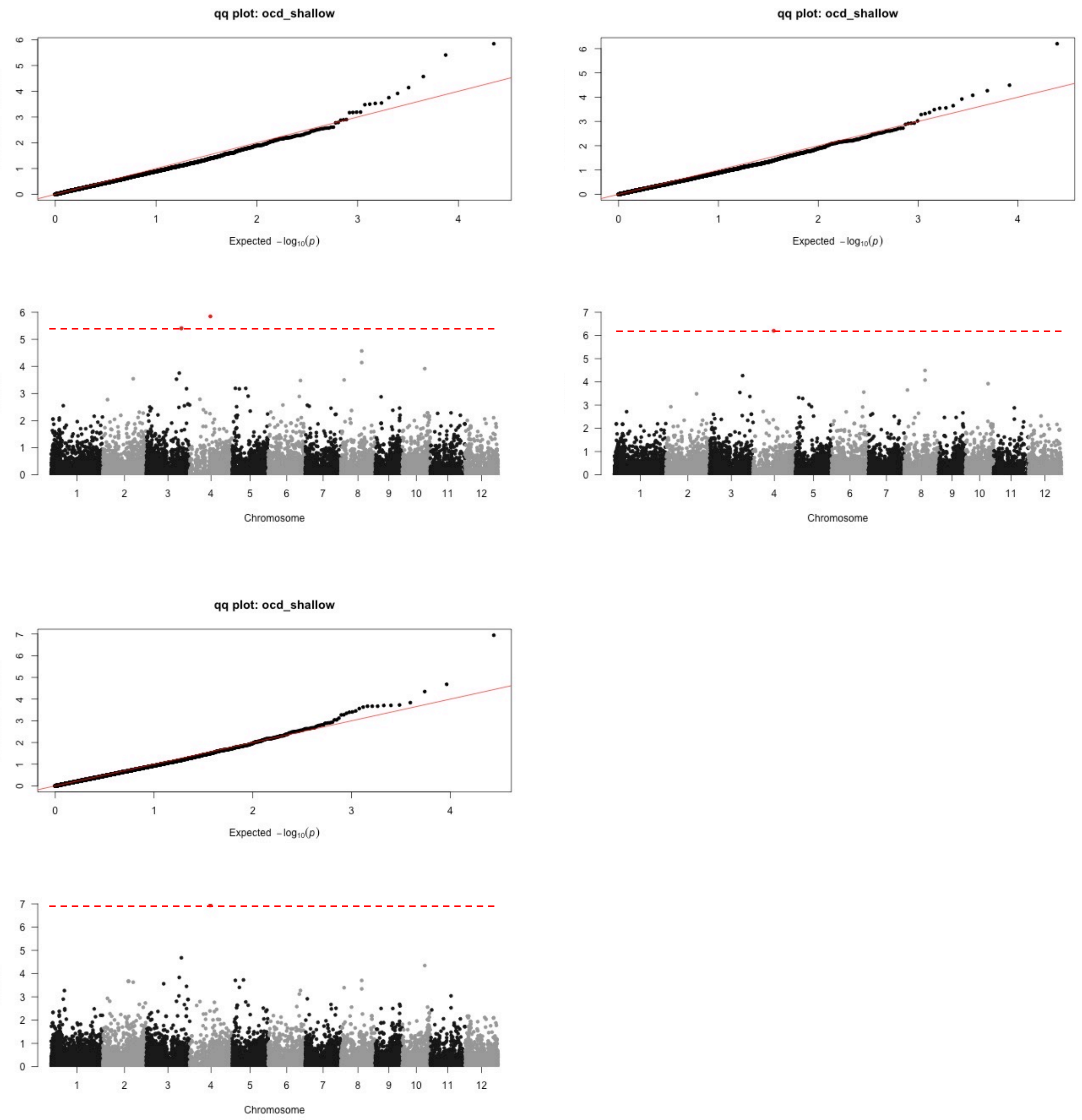

Figure S8. Genome-wide association results of organic carbon density (topsoil) in the small, medium, and full panels. From top-left to top-right to bottom-left : small, medium, and full. Shown are the quantile-quantile plot (top) and manhattan plot (bottom). Significant SNPs at False Discovery Rate threshold of 0.05 (Benjamini-Hochberg method) are annotated in red and a dashed red horizontal line is drawn at the level of the least significant SNP.

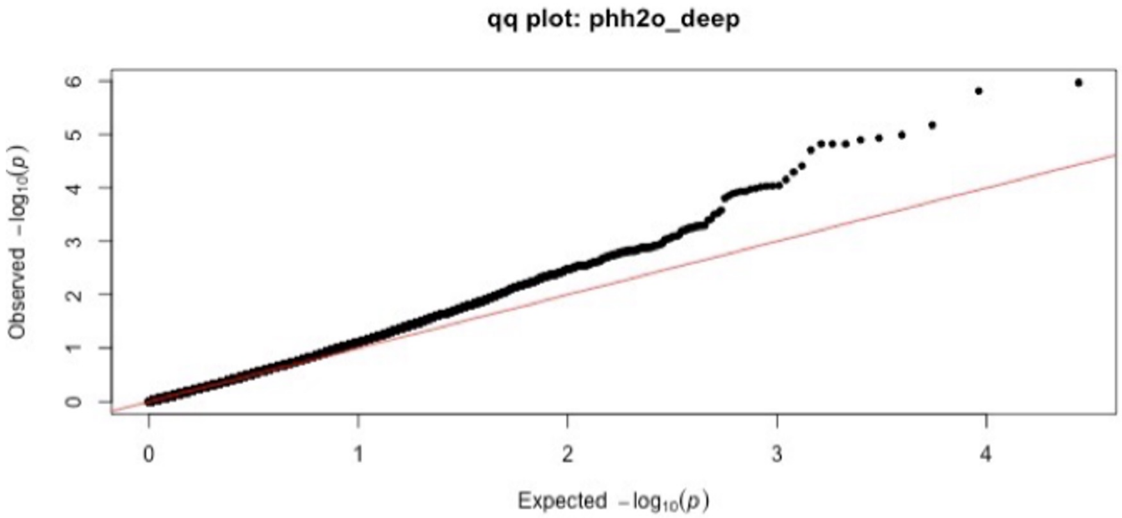

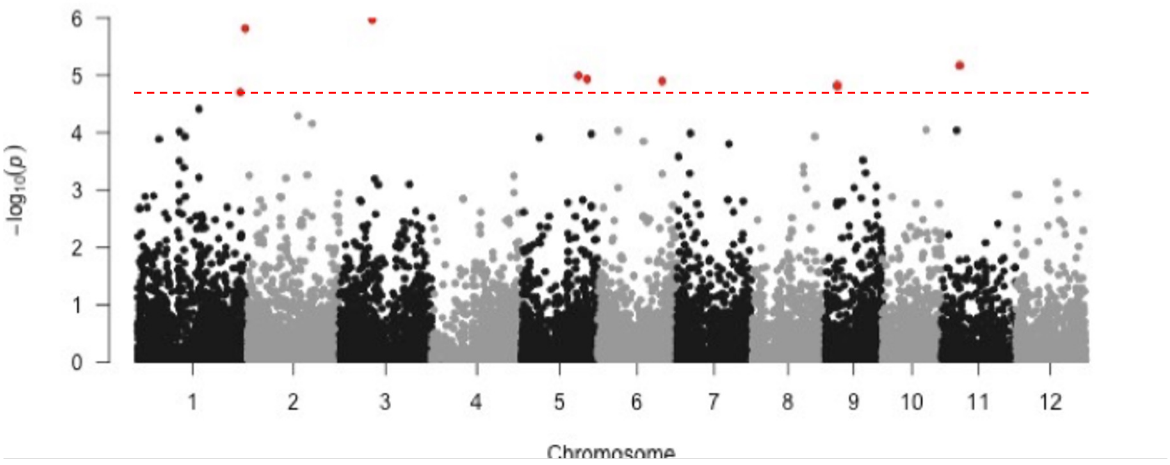

Figure S9. Genome-wide association results of soil pH (subsoil) in the medium panel. Shown are the quantile-quantile plot (top) and manhattan plot (bottom). Significant SNPs at False Discovery Rate threshold of 0.05 (Benjamini-Hochberg method) are annotated in red and a dashed red horizontal line is drawn at the level of the least significant SNP.

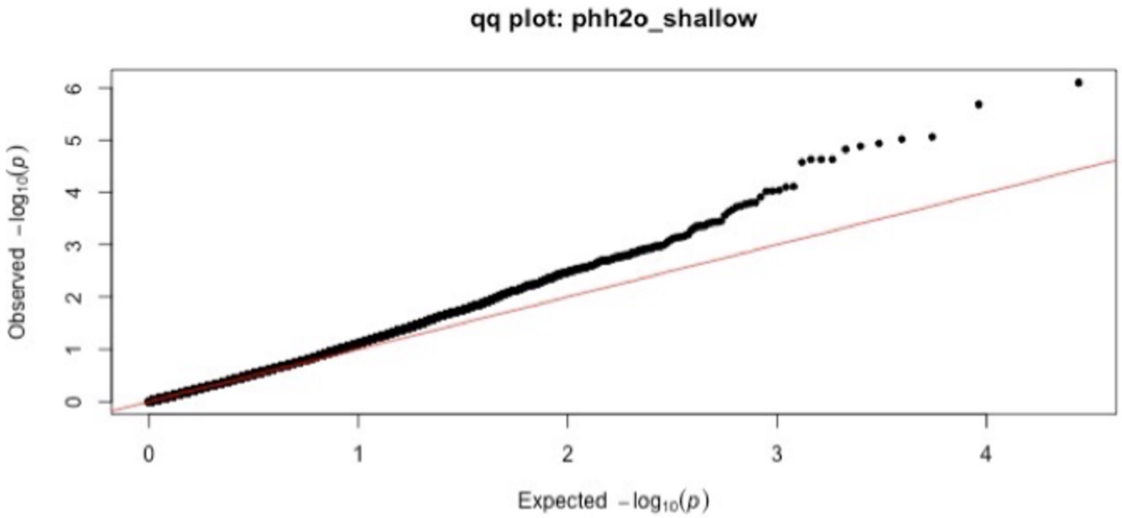

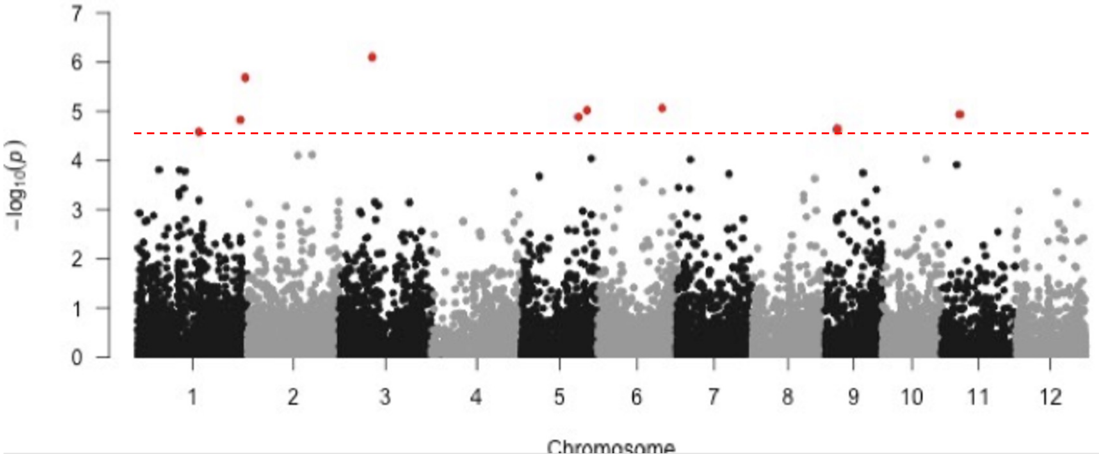

Figure S10. Genome-wide association results of soil pH (topsoil) in the medium panel. Shown are the quantile-quantile plot (top) and manhattan plot (bottom). Significant SNPs at False Discovery Rate threshold of 0.05 (Benjamini-Hochberg method) are annotated in red and a dashed red horizontal line is drawn at the level of the least significant SNP.

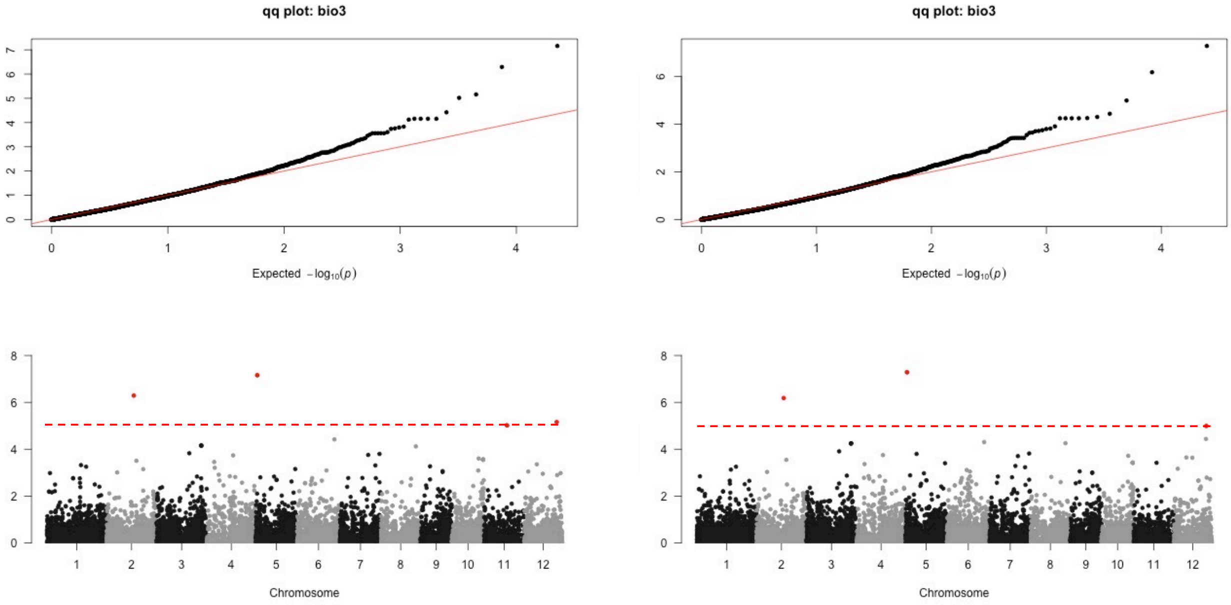

Figure S11. Genome-wide association results of bio3 (isothermality) in the small and medium panels. From left to right: small and medium. Shown are the quantile-quantile plot (top) and manhattan plot (bottom). Significant SNPs at False Discovery Rate threshold of 0.05 (Benjamini-Hochberg method) are annotated in red and a dashed red horizontal line is drawn at the level of the least significant SNP and a dashed red horizontal line is drawn at the level of the least significant SNP.

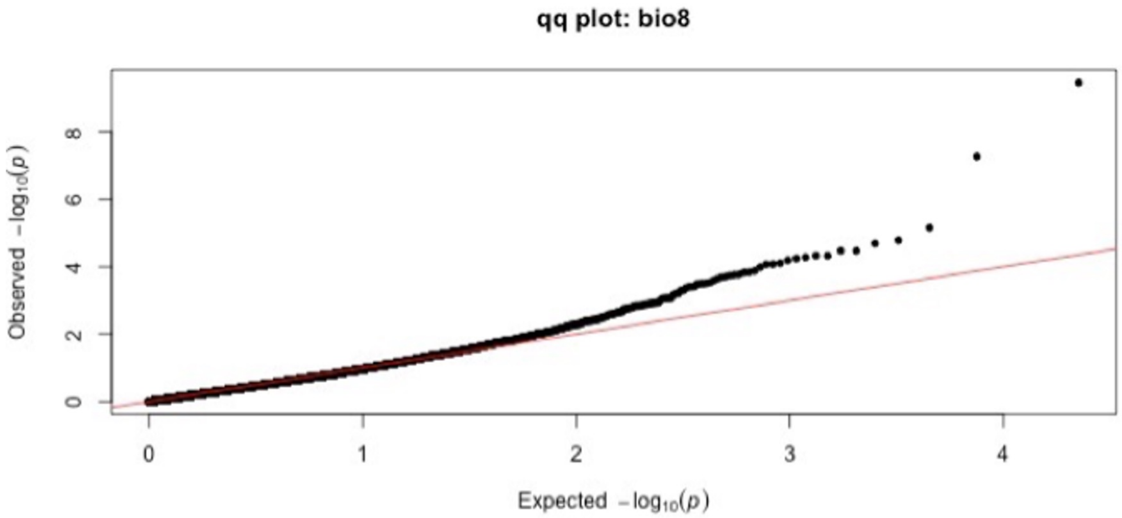

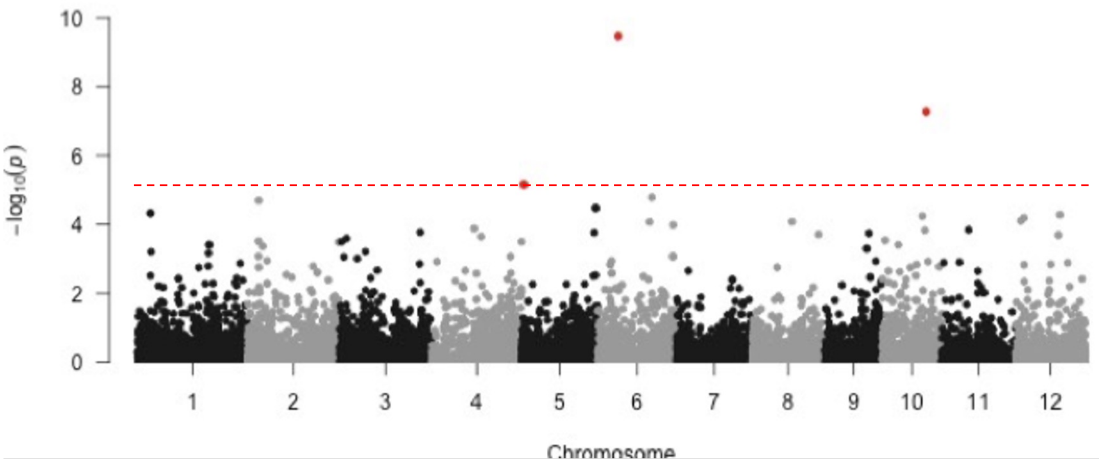

Figure S12. Genome-wide association results of bio8 (mean temperature of warmest quarter) in the medium panel. Shown are the quantile-quantile plot (top) and manhattan plot (bottom). Significant SNPs at False Discovery Rate threshold of 0.05 (Benjamini-Hochberg method) are annotated in red and a dashed red horizontal line is drawn at the level of the least significant SNP.

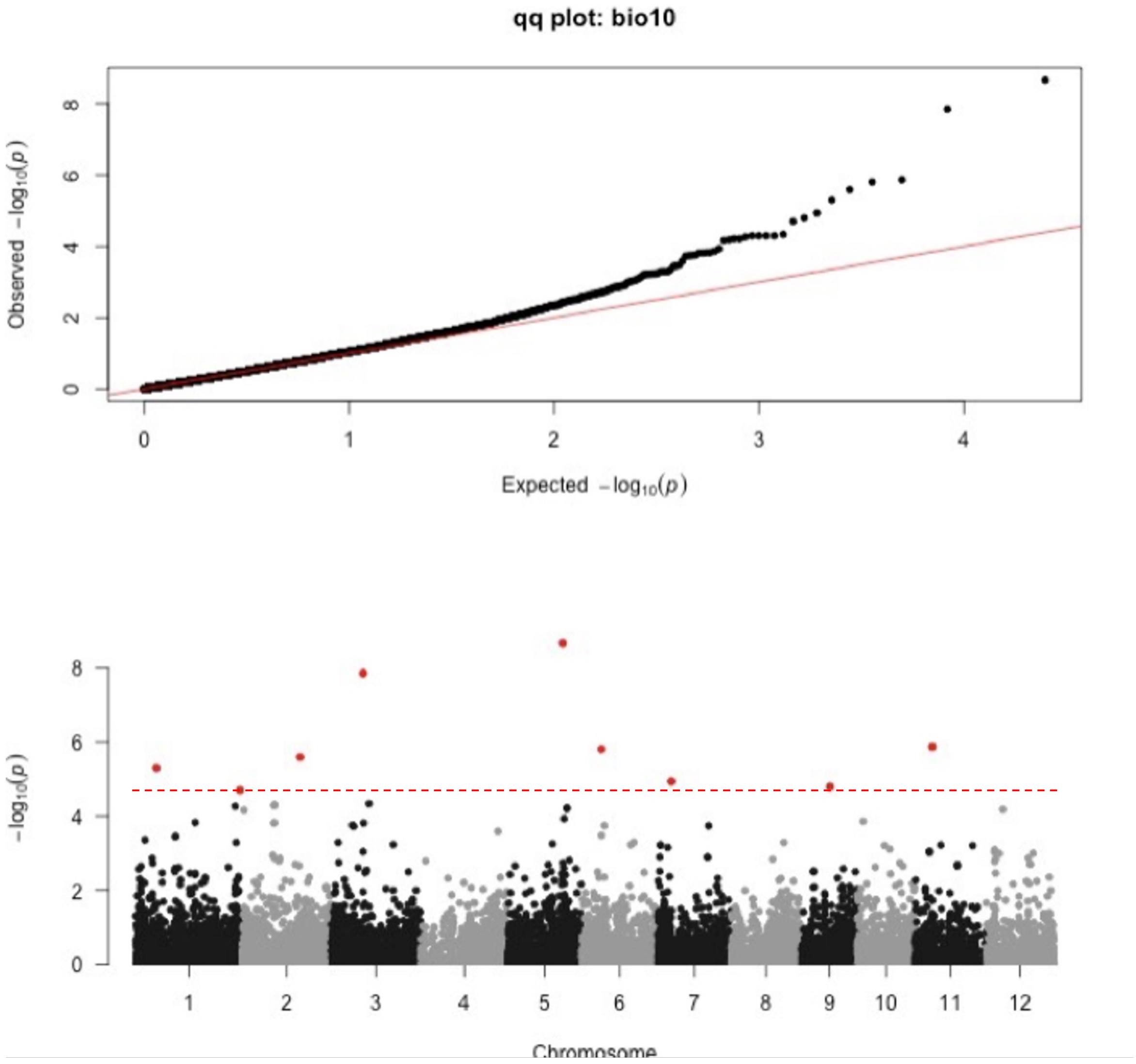

Figure S13. Genome-wide association results of bio10 (mean temperature of warmest quarter) in the medium panel. Shown are the quantile-quantile plot (top) and manhattan plot (bottom). Significant SNPs at False Discovery Rate threshold of 0.05 (Benjamini-Hochberg method) are annotated in red and a dashed red horizontal line is drawn at the level of the least significant SNP.

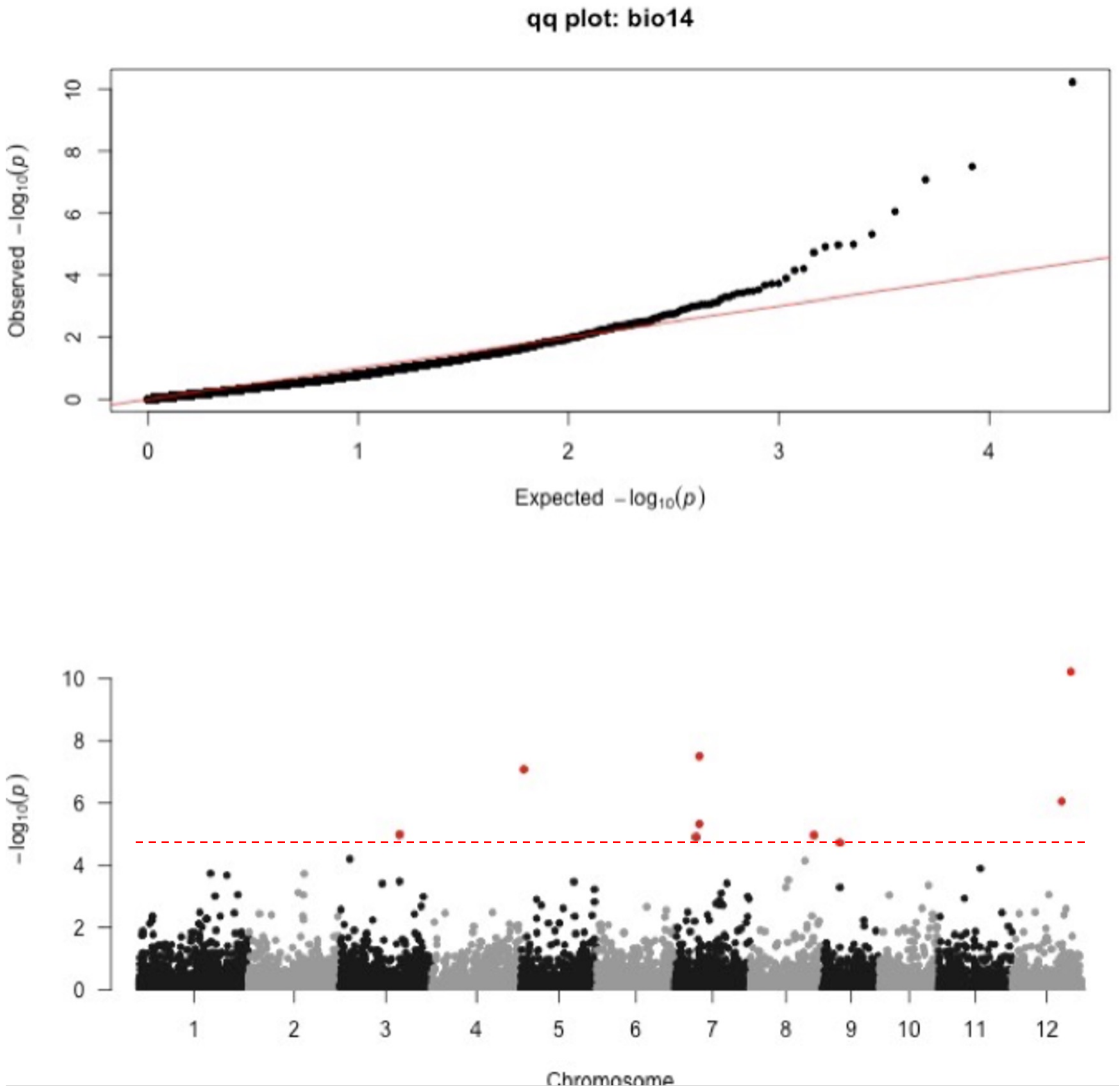

Figure S14. Genome-wide association results of bio14 (precipitation in the driest month) in the medium panel. Shown are the quantile-quantile plot (top) and manhattan plot (bottom). Significant SNPs at False Discovery Rate threshold of 0.05 (Benjamini-Hochberg method) are annotated in red and a dashed red horizontal line is drawn at the level of the least significant SNP.

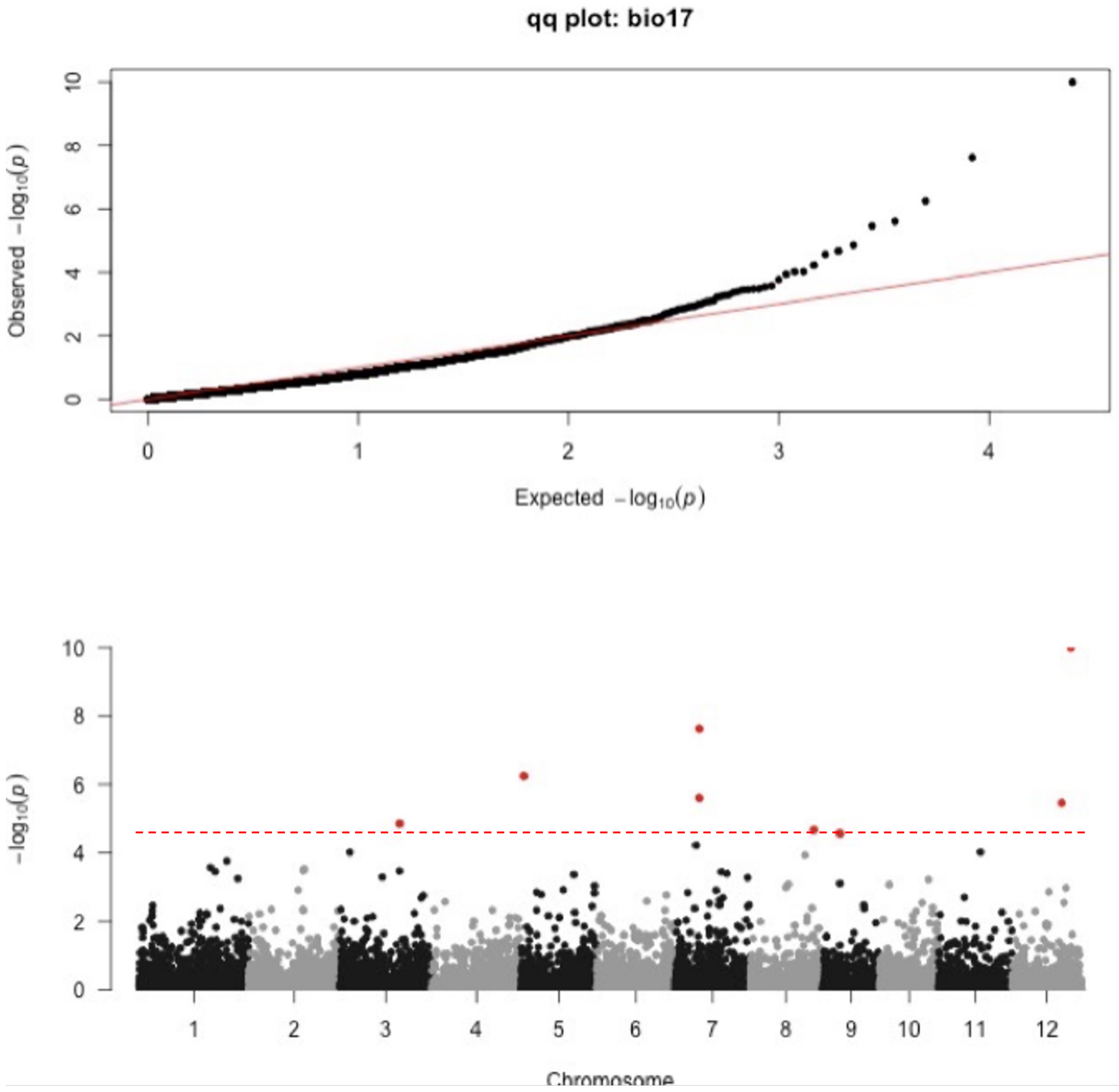

Figure S15. Genome-wide association results of bio17 (precipitation in the driest quarter) in the medium panel. Shown are the quantile-quantile plot (top) and manhattan plot (bottom). Significant SNPs at False Discovery Rate threshold of 0.05 (Benjamini-Hochberg method) are annotated in red and a dashed red horizontal line is drawn at the level of the least significant SNP.

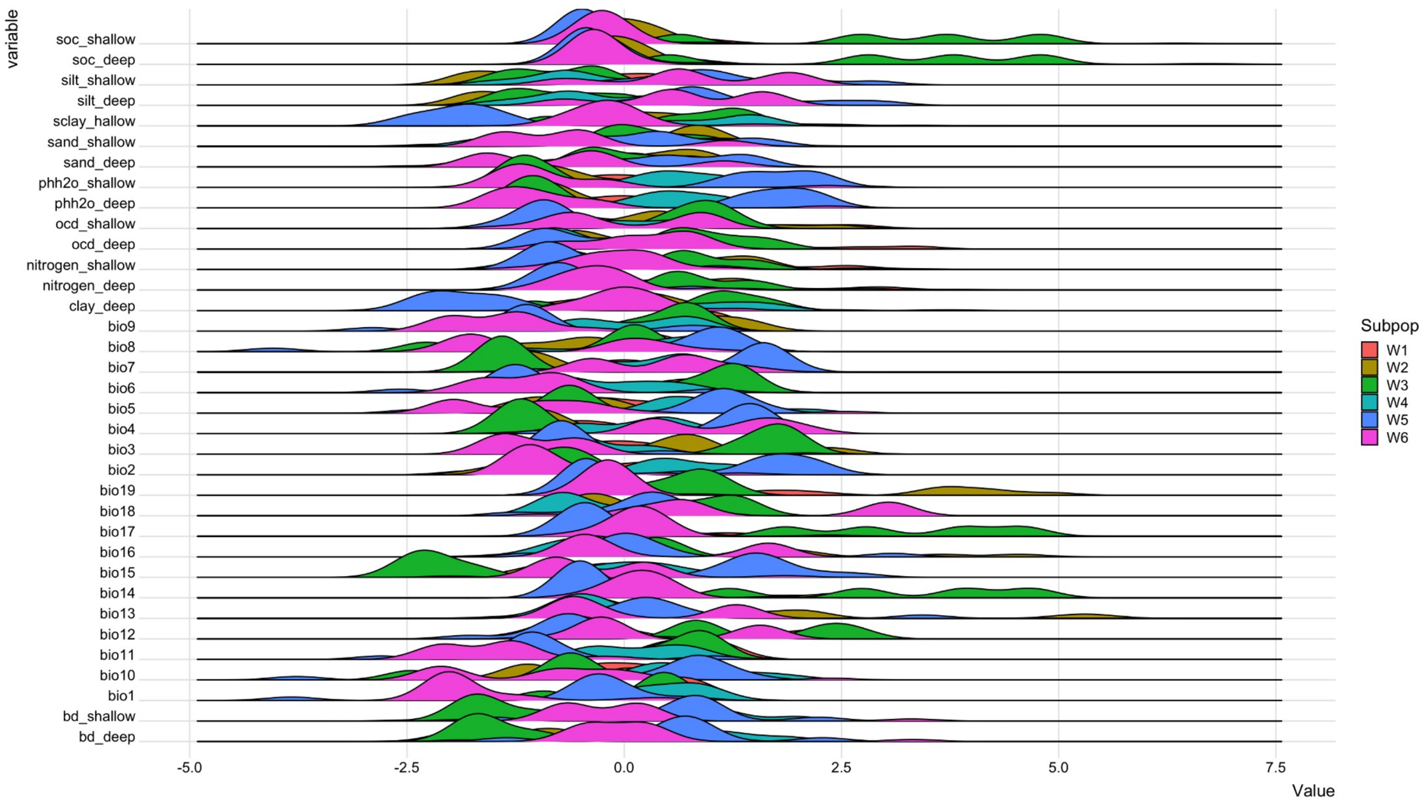

Figure S16. Univariate distributions of environmental variables across wild subpopulations. Values are standardized to a mean of 0 and standard deviation of 1. Here the density of the distributions of each subpopulations indicated by colors. Admixed individuals identified by Kim et al., 2016 are not shown here.
